# Epstein–Barr virus-associated B-cell lymphoproliferative disorder meeting the definition of CAEBV B cell disease: a case report

**DOI:** 10.1186/s12879-023-08430-6

**Published:** 2023-07-07

**Authors:** Yaxian Ma, Yuhan Bao, Miao Zheng

**Affiliations:** 1 Department of Hematology, Tongji Hospital, Tongji Medical College, Huazhong University of Science and Technology, Wuhan, China; 2 Immunotherapy Research Center for Hematologic Diseases of Hubei Province, Wuhan, China

**Keywords:** Chronic active Epstein-Barr virus infection, Lymphoproliferative disorder, B cell, PIK3CD, Missense mutation

## Abstract

**Background:**

Chronic active Epstein-Barr virus infection (CAEBV) is a systemic EBV-positive lymphoproliferative disorder (EBV-LPD) considered to be associated with a genetic immunological abnormality, although its cause is still unclear. EBV is usually detected in T cells or NK cells in CAEBV patients with only a few cases involving B cells described in East Asia, which may be due to differences in genetic and environmental factors.

**Case description:**

A 16-year-old boy who seemed to be diagnosed as CAEBV of B cell type was studied. The patient had IM-like symptoms persisting for more than 3 months, high levels of EBV DNA in the PB, and positive EBER in situ hybridization in B cells. In addition, to exclude underlying genetic disorders, we performed next-generation sequencing (NGS) and whole-exome sequencing (WES), which identified the missense mutation in PIK3CD (E1021K), ADA (S85L) and CD3D (Q140K) in the patient while no same genetic mutation was detected in his parents and sister. However, there is no diagnosis of CAEBV of B cell type in the most recent World Health Organization classification of tumors of hematopoietic and lymphoid tissues, therefore we finally diagnosed this patient as EBV-B-LPD.

**Conclusions:**

This study shows a rare case of a patient meeting the definition of CAEBV B-cell disease in East Asia. Meanwhile, the case indicates that the missense mutation and the disease are related.

## Background

Epstein–Barr virus (EBV), herpes Virus 4 is a ubiquitous linear double-stranded DNA virus carried by approximately 95% of the adult population worldwide. Most primary infections occur during childhood and are usually asymptomatic, but sometimes infection occurs in adolescents or young adults results in infectious mononucleosis (IM), which typically manifests as fever, pharyngitis, lymphadenopathy, hepatosplenomegaly and atypical lymphocytosis [1, 2]. Although in most cases, these manifestations of IM resolve spontaneously without sequelae, rare persons may develop a life-threatening syndrome called chronic active EBV infection (CAEBV) [3].

CAEBV is a systemic EBV-positive lymphoproliferative disorder characterized by infectious mononucleosis-like symptoms lasting for at least 3 months and high levels of blood EBV DNA in immunocompetent persons [3–5]. Although various therapies have been used to relieve the symptoms, only hematopoietic stem cell transplantation (HSCT) is regarded as the only curative therapy [6, 7].

Most CAEBV cases to date have been reported in East Asia, by comparison, CAEBV is much rarer in Western countries. In addition, CAEBV patients in the United States most often show the infection involving B or T cells [8], while in East Asia, the cases involving T or NK cells have commonly been observed with only a few cases involving B cells described [9]. This condition is thought to be due to differences in genetic and environmental factors, but the pathophysiology and specific mechanisms are still unclear.

On the other hand, CAEBV of B-cell type is not included in the Classification of Neoplastic Diseases of the Hematopoietic and Lymphoid Tissues World Health Organization (WHO) in 2016, while they defined CAEBV of T/NK-cell type as EBV-positive T/NK-cell neoplasms [5, 10]. CAEBV of B-cell type is classified as Epstein–Barr virus-associated B-cell lymphoproliferative disorder (EBV-B-LPD), which happened when EBV induces B-cell transformation, and the balance between the EBV and host immune system is disrupted [11, 12]. EBV-B-LPDs represent a broad range of clinicopathology, from indolent and self-limited diseases to aggressive lymphoma [12–15].

Here, we describe a case of a patient diagnosed with Epstein–Barr virus-associated B-cell lymphoproliferative disorder with gene mutation, which was like CAEBV of B-cell type.

## Case presentation

The key clinical events of this patient are depicted in Table 1.

**Table 1:** Brief chronology of the key clinical course in the present case

Date	Key clinical course
2020/3	Sinusitis, secretory otitis media, mixed hearing loss and cervical lymph nodes swelling
	Biopsy of nasopharynx and cervical lymph node suggesting EBV-associated lymphoproliferative disorder
2020/8	Stomach discomfort
2021/1	Edema of both lower limbs, lung infection and hypoproteinemia
	Diagnosis of CAEBV-associated B-cell lymphoproliferative diseases (Grade 2–3)
	Discovery of the missense mutation in PIK3CD (E1021K), ADA (S85L) and CD3D (Q140K)
	2 cycles of R-CVP
2021/7	Multiple organ failure and death

The patient was a previously healthy 16-year-old boy with no personal or family history of immunodeficiency. He came to our hospital because of cervical lymph nodes swelling for five months. Five months ago, he was considered for sinusitis, secretory otitis media and mixed hearing loss in March 2020. Subsequent MRI of the nasopharynx showed obvious thickening of the nasopharyngeal cavity sidewall and cervical multiple lymph nodes. Then he underwent a biopsy of the nasal cavity and nasopharynx, which showed EBV-associated lymphoproliferative lesions with B cell proliferation predominance. Immunohistology of his biopsy revealed positive EBV-encoded small RNA (EBER). An assay for gene rearrangement showed clonal rearrangement of B cell receptors, with no clonal rearrangement of T cell receptors. In May 2020, a biopsy of the cervical lymph nodes was performed, which also showed EBV-associated lymphoproliferative disorder prone to IM. In August 2020, the patients developed stomach discomfort, and a subsequent gastroscopy showed gastric mass and duodenal lesions. Later pathology demonstrated that gastric mass and duodenal lesions conformed to lymphoid tissue proliferative lesions. After entering our hospital, bone marrow puncture and bone marrow biopsy showed no obvious abnormality of lymphocytes.

On January 6, 2021, the patient was re-admitted to the hospital due to edema of both lower limbs. In addition, the patient developed moderate anemia, lung infection, hypoproteinemia and bilateral middle ear mastoiditis. Ultrasound examination showed hepatosplenomegaly, abdominal effusion and multiple lymphadenopathies on both sides of the neck, axilla and groin. The diagnosis of second electronic gastroscopy was that the esophagus, gastric body, gastric antrum and duodenal bulb had multiple bulges. The peripheral blood (PB) level of EBV-DNA was positive (5.02 × 10^3^ copies/mL), and EBV DNA level in plasma was less than 500 copies/mL. For detecting the phenotypes of the infected cells, we perform a combined analysis of magnetic bead sorting and PCR for EBV DNA, which demonstrated that EBV was detected in B cells (3.373 × 10^4^ copies/2 × 10^5^cells), NK cells (4.912 × 10^2^ copies/2 × 10^5^cells), PBMC cells (1.629 × 10^4^ copies/2 × 10^5^cells), and undetected in T cells. Therefore, B cell was the EBV-predominant cell type in this case.

**Fig. 1:**
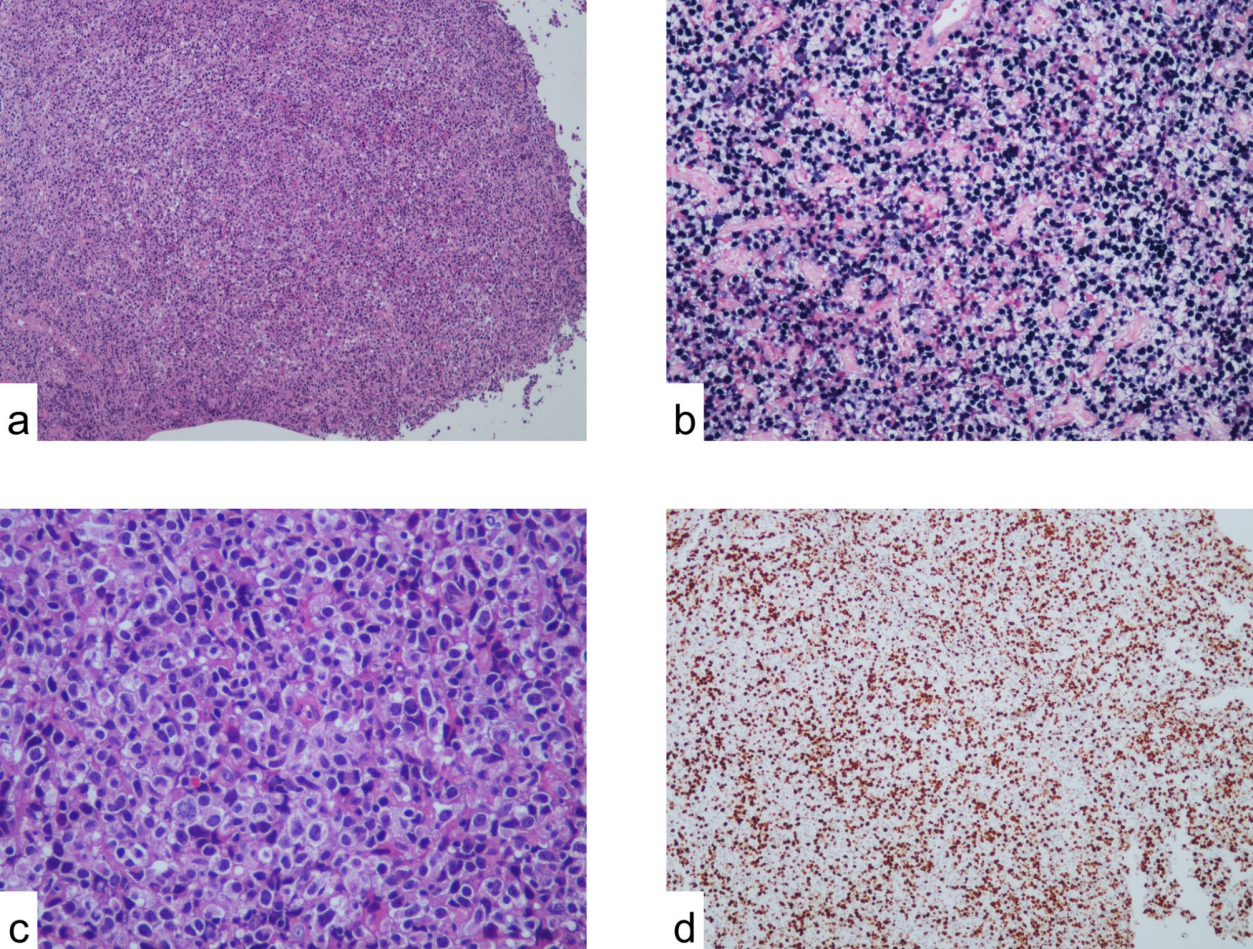
Histologic images of the gastric mass. (**a**) Diffuse lymphocyte components in the mucosa as viewed with hematoxylin and eosin staining (magnification ×100). (**b**) Diffuse B lymphocytes and plasma cells as viewed with hematoxylin and eosin staining (magnification ×400). (**c**) Epstein–Barr virus (EBV) + cells as viewed with in situ hybridization (magnification ×200). (**d**) Immunohistochemistry showing 80% of the B cells were Ki-67+ (magnification ×100)

**Fig. 2:**
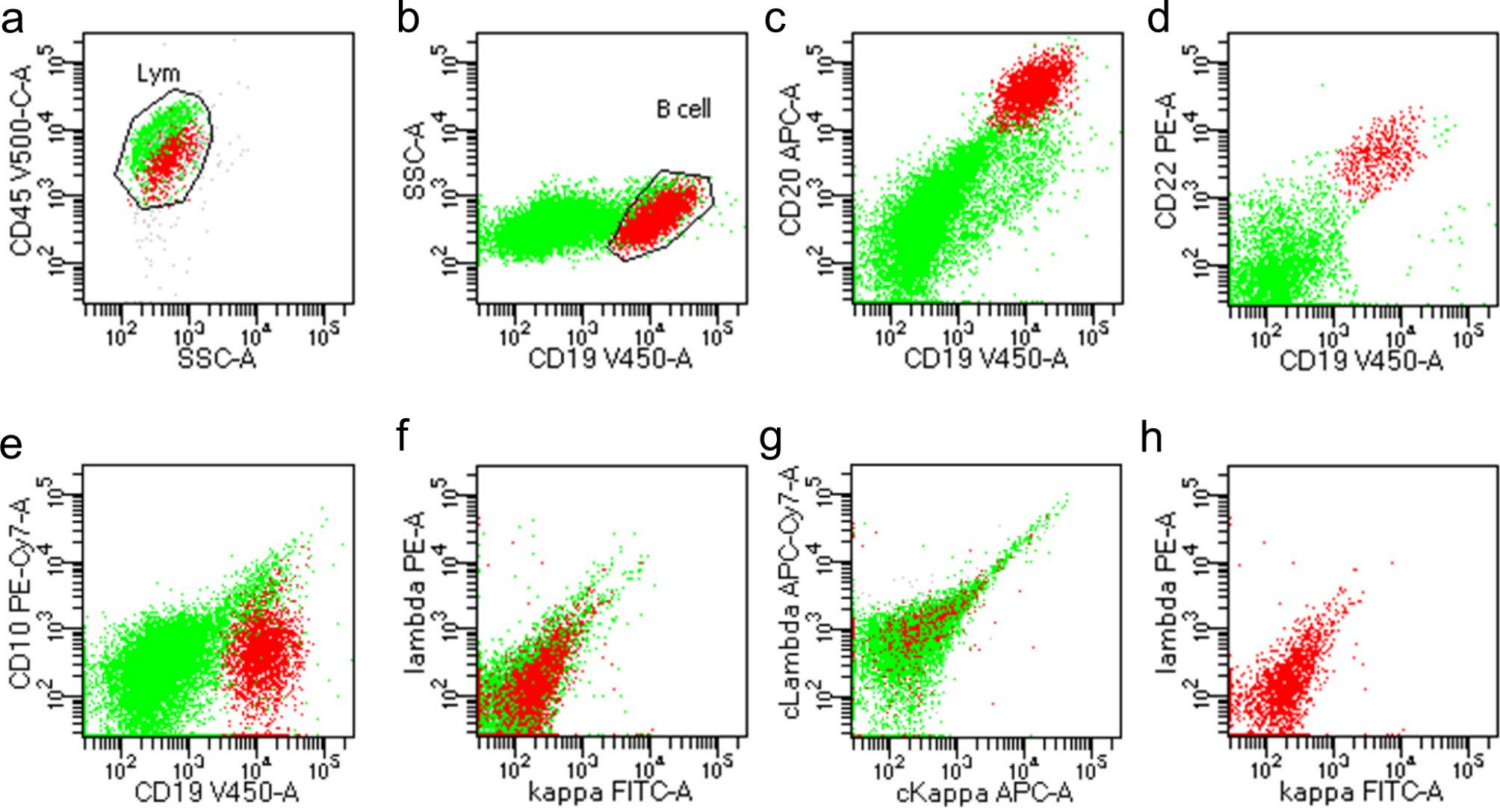
Flow cytometry analysis of the gastric mass. (**a**) In puncture tissue samples, lymphocytes accounted for about 93.5% of all nucleated cells. (**b–h**) Abnormal cells expressing CD19, CD20, CD22 and not expressing CD10, kappa, lambda, ckappa, clambda were considered as monoclonal abnormal mature B lymphocytes

In order to confirm the diagnosis, the patient underwent a second gastric and duodenal biopsy and further evaluation with immunohistochemical and gene rearrangement studies, which revealed CAEBV-associated B-cell lymphoproliferative diseases (Grade 2–3). The result of the immunohistochemical study was as follows: B cells were positive for CD19, CD20, CD22, PAX5, CD79b, MUM, weakly positive for C-MYC, BCL2, BCL6, κ, λ, and negative for CD10, CyclinD1, CD21, MNDA, LEFl, TdT. The Ki-67 index was about 80%; Background T cells were positive for CD3, CD4, CD5, CD8, CD43 (CD8 (+) > CD4 (+)), weakly positive for EBNA2, and negative for ALK, CD56; Both B cells and T cells were positive for EBER In situ hybridization in the patient’s gastric lesions (diffuse +, about 300 positive cells/HPFs), with B cells predominantly (Fig. 1). The flow cytometry showed monoclonal abnormal mature B lymphocyte negative for CD5, CD10, and the Ki-67 index was 21.6% (Fig. 2).

After being diagnosed with CAEBV-associated B-cell lymphoproliferative diseases (Grade 2–3), he received two cycles of R-CVP (rituximab, cyclophosphamide, vincristine and prednisolone), which temporarily controlled the progression of CAEBV and the patient’s symptoms improved.

In February 2021, to exclude underlying genetic disorders, we performed next-generation sequencing (NGS). Whole-exome sequencing (WES) identified three missense mutations in the patient, including PIK3CD (E1021K) with 51.2% mutation abundance, ADA (S85L) with 50.0% mutation abundance and CD3D (Q140K) with 52.2% mutation abundance. The patient and his family members were recommended by doctors for allogeneic stem cell transplantation. Therefore, they were screened for related gene mutations, while no same genetic mutation was detected in his parents and sister. Unfortunately, his infection worsened while waiting for HSCT, he eventually fell into the stage of multiple organ failure and died in July.

## Discussion and conclusions

EBV is the first confirmed human tumor virus, which is etiologically linked to two lymphoproliferative diseases (B-LPD and T/NK-LPD) and a wide range of distinct human tumors [16]. According to the most recent WHO classification of tumors of hematopoietic and lymphoid tissues, CAEBV is one of the representative T/NK-cell EBV-associated lymphoproliferative disorders (LPDs). Its diagnostic criteria include IM-like symptoms lasting for at least 3 months, elevated EBV DNA load (more than 10^2.5^ copies/µg DNA) in the PB or the tissue lesion, demonstration of EBV infection in the affected tissues in patients without other possible diagnoses (IM, autoimmune diseases, malignancy, immunodeficiency, and other underlying diseases with potential immunosuppression) [3, 5].

In the case we reported, this patient’s disease shares many features of T/NK-cell CAEBV. He had IM-like symptoms persisting for more than 3 months and high levels of EBV DNA in the PB. The analysis of the phenotypes of the infected cells through immune histological staining, EBV sorting PCR and EBER in situ hybridization showed that the infection involved B cells. It is important to exclude known causes of immunodeficiency, so we performed NGS. Although the result identified three missense mutations in the patient, the normal immune function before onset and immunoglobulin levels in the normal range suggested that this mutation did not cause primary immunodeficiency (PID).

According to the diagnostic criteria of CAEBV of NK/T cell type, this patient seemed to be diagnosed as CAEBV of B cell type. CAEBV patients in East Asia most often show the infection involving NK or T cells with only a few cases involving B cells described [9], which may be due to differences in genetic and environmental factors. However, as there is no diagnosis of CAEBV of B cell type in the most recent WHO classification of tumors of hematopoietic and lymphoid tissues [5, 13], we finally diagnosed this patient as EBV-B-LPD and the etiology may be related to the missense mutation in PIK3CD (E1021K), ADA (S85L) and CD3D (Q140K).

Current studies have not found a consistent cause of CAEBV disease, which may be related to genetic etiology. It has been reported that somatic mutations of infected cells and genetic mutations of EBV are involved in the development of CAEBV. Several gene mutations in patients meeting the definition of CAEBV B-cell disease have been reported, including gene mutations in perforin [17], Munc13-4 [18], Munc 18 − 2 [18, 19], MAGT1 [20], GATA2 [21], PIK3CD [22] and CTPS1 [23].

The PIK3CD gene encodes the catalytic subunit phosphatidylinositol-4, 5-bisphosphate 3-kinase catalytic subunit delta isoform (p110δ), which together form the heterodimeric protein phosphoinositide-3-kinase δ (PI3Kδ) with a p85 family regulatory subunit [24]. PI3Kδ suppresses forkhead box O1 (FOXO1) and transcription promotes the activation of mechanistic target of rapamycin (mTOR) through phosphoinositide-dependent kinase 1 (PDK1) and AKT, which act in concert to phosphorylate substrates [24]. The PI3K/AKT/mTOR pathway plays an important role in controlling the proliferation, activation and survival of tumor cells [24].

The GLU1021LYS (E1021K) mutation resulting in a gain of function is the most frequent mutation in PIK3CD, which can lead to increased activity of the encoded PI3Kδ [24–27]. It is believed that heightened PI3Kδ signal stimulates abnormal development and proliferation of leukocytes and causes susceptibility to lymphoma [24–27]. Besides that, enhanced intrinsic PI3K signal may drive CD8^+^ T-cell terminal differentiation, premature exhaustion and immunosenescence, which might contribute to impaired control of EBV [28].

Mutations in the ADA gene will lead to Adenosine deaminase (ADA) deficiency, a severe combined immunodeficiency (SCID), which is a rare inherited disorder of purine metabolism characterized by abnormalities of immune system development and function [29]. The CD3D gene encodes the CD3δ polypeptide, which participates in the formation of the TCR-CD3 complex and plays an important role in T cell development and signal transduction [30]. Abnormal CD3D gene may lead to CD3D deficiency-related T cell immunodeficiency. The missense mutation in ADA (S85L) and CD3D (Q140K) is of unknown significance, possibly associated with immunodeficiency.

In summary, in this study, we demonstrated a rare case of a patient with EBV-B-LPD sharing many features of CAEBV in East Asia. Our case also indicated there may be a relationship between the missense mutation and the disease, but the pathophysiology and specific mechanisms are still unclear. This prompts us to collect more clinical data and design a more detailed clinical research to more clearly define the EBV-B-LPD very similar to CAEBV, and further clarify the mechanism of EBV-B-LPD.

## Data Availability

The datasets generated during and/or analysed during the current study are available from the corresponding author on reasonable request.

## Abbreviations

APDS: Activated phosphoinositide 3- kinase delta syndrome
EBV: Epstein-Barr virus
CAEBV: Chronic active EBV infection
PID: Primary immunodeficiency
PI3Kδ: Phosphoinositide 3- kinaseδ
LPD: Lymphoproliferative disorder
IM: Infectious mononucleosis
EBV-B-LPDs: EBV-associated B-cell lymphoproliferative disorders
EBER: EBV-encoded small RNA
PB: Peripheral blood
NGS: Next-generation sequencing
WES: Whole-exome sequencing
FOXO1: Forkhead box O1
mTOR: Mechanistic target of rapamycin
PDK1: Phosphoinositide-dependent kinase 1
